# Supplementary material for: A tutorial on individualized treatment effect prediction from randomized trials with a binary endpoint
Source: Stat Med. 2021 Aug 16;40(26):5961–81. doi: 10.1002/sim.9154 (PMC9291969; doi:10.1002/sim.9154)
Supplement: Supplementary file 1 — Appendix S1 Online Supporting Material [file SIM-40-5961-s001.zip › CalibrationFigures.pdf]

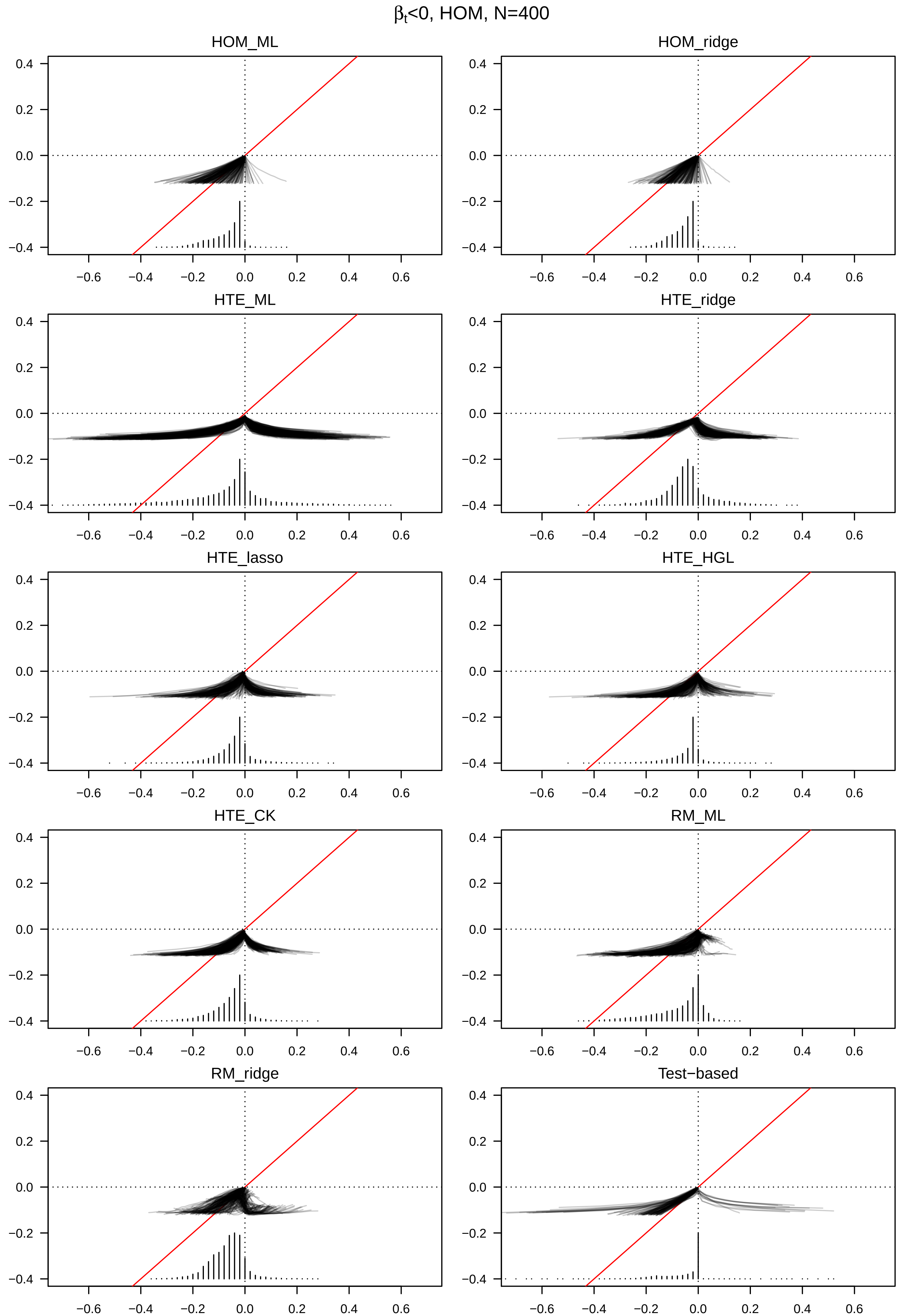

βt<0, HOM, N=400
HOM_ML
HOM_ridge
HTE_ML
HTE_ridge
HTE_lasso
HTE_HGL
HTE_CK
RM_ML
RM_ridge
Test−based
0.4
0.2
0.0
−0.2
−0.4
−0.6
0.6

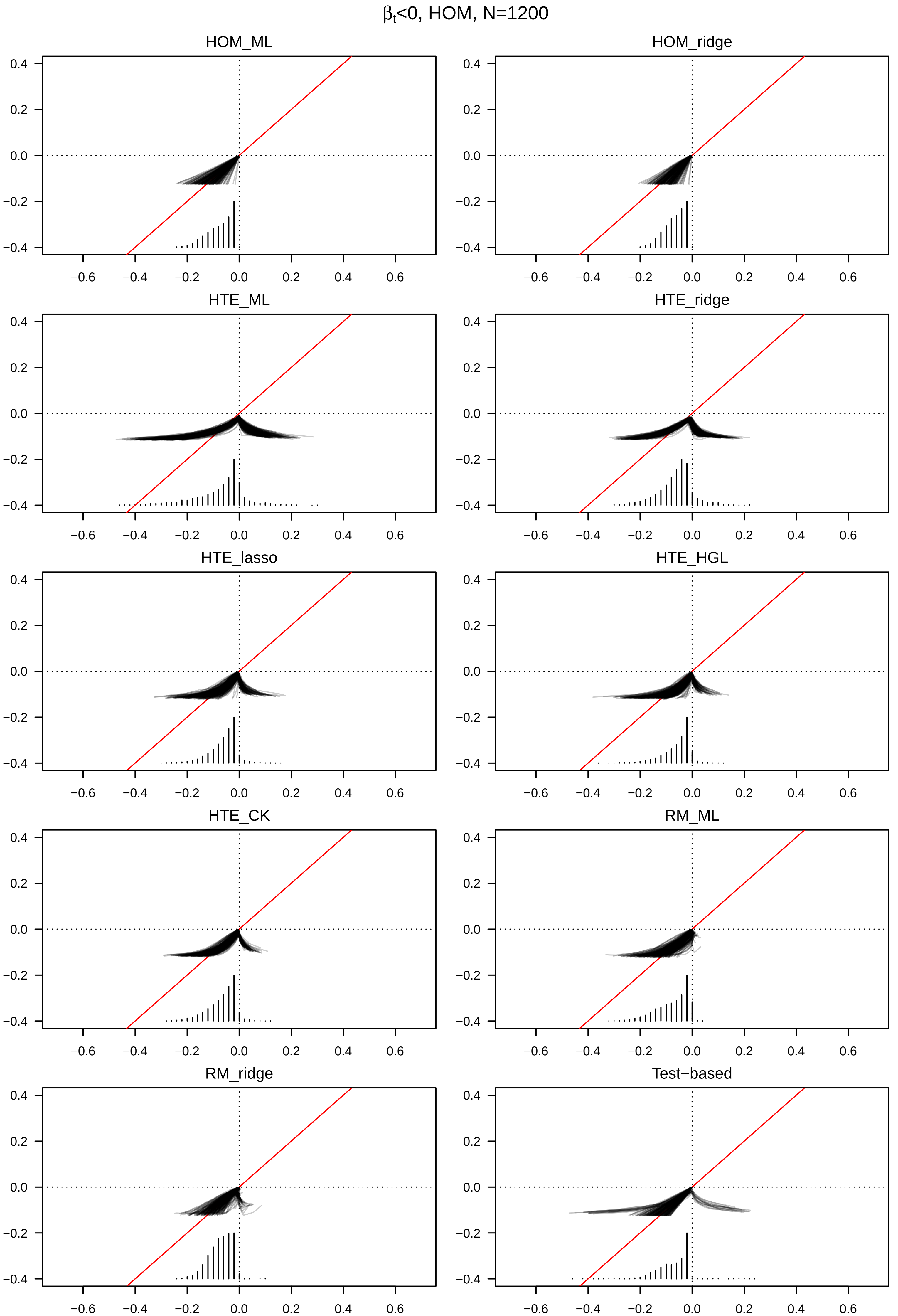

β_t<0, HOM, N=1200
HOM_ML
HOM_ridge
HTE_ML
HTE_ridge
HTE_lasso
HTE_HGL
HTE_CK
RM_ML
RM_ridge
Test−based

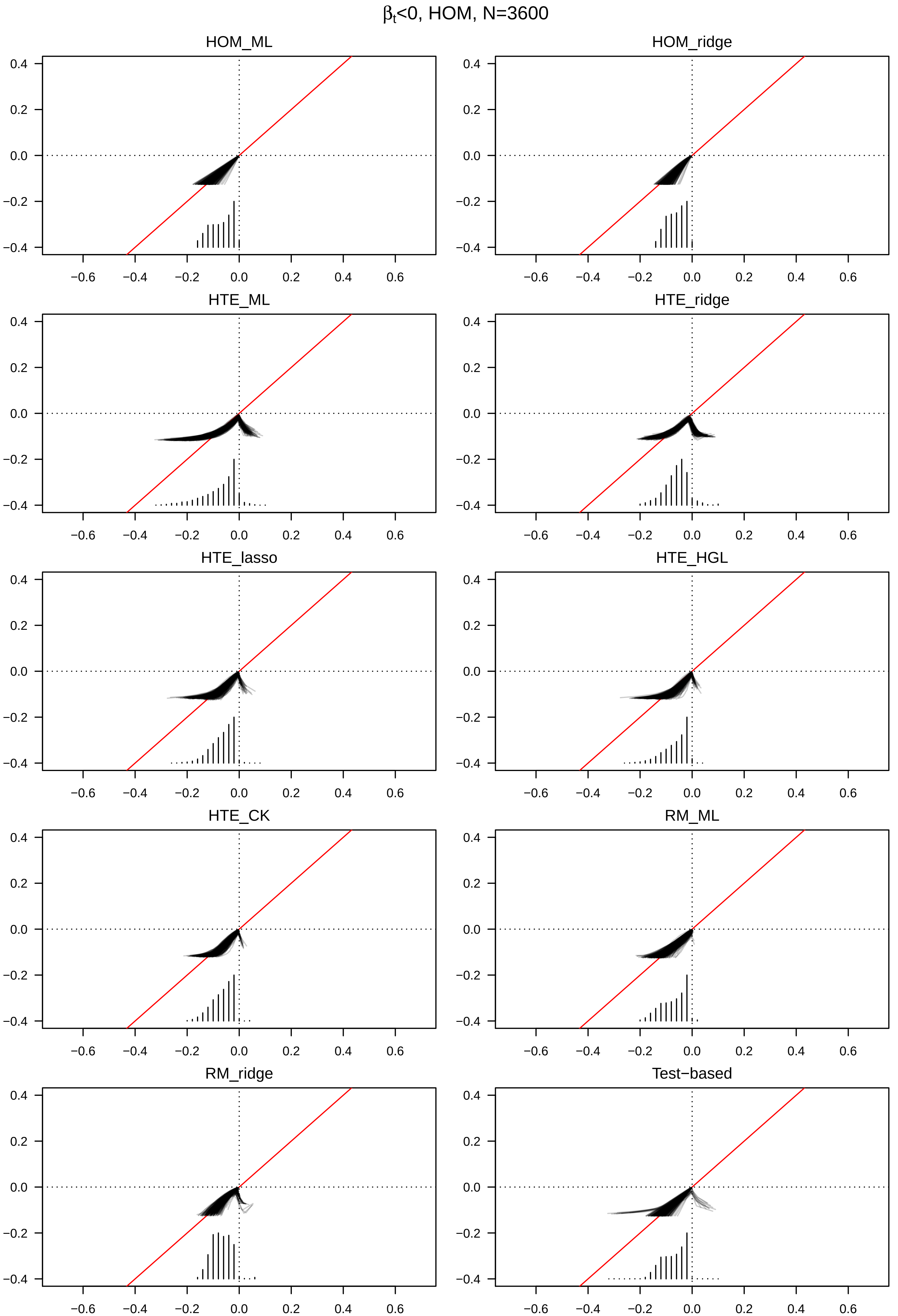

βt<0, HOM, N=3600
HOM_ML
HOM_ridge
HTE_ML
HTE_ridge
HTE_lasso
HTE_HGL
HTE_CK
RM_ML
RM_ridge
Test−based

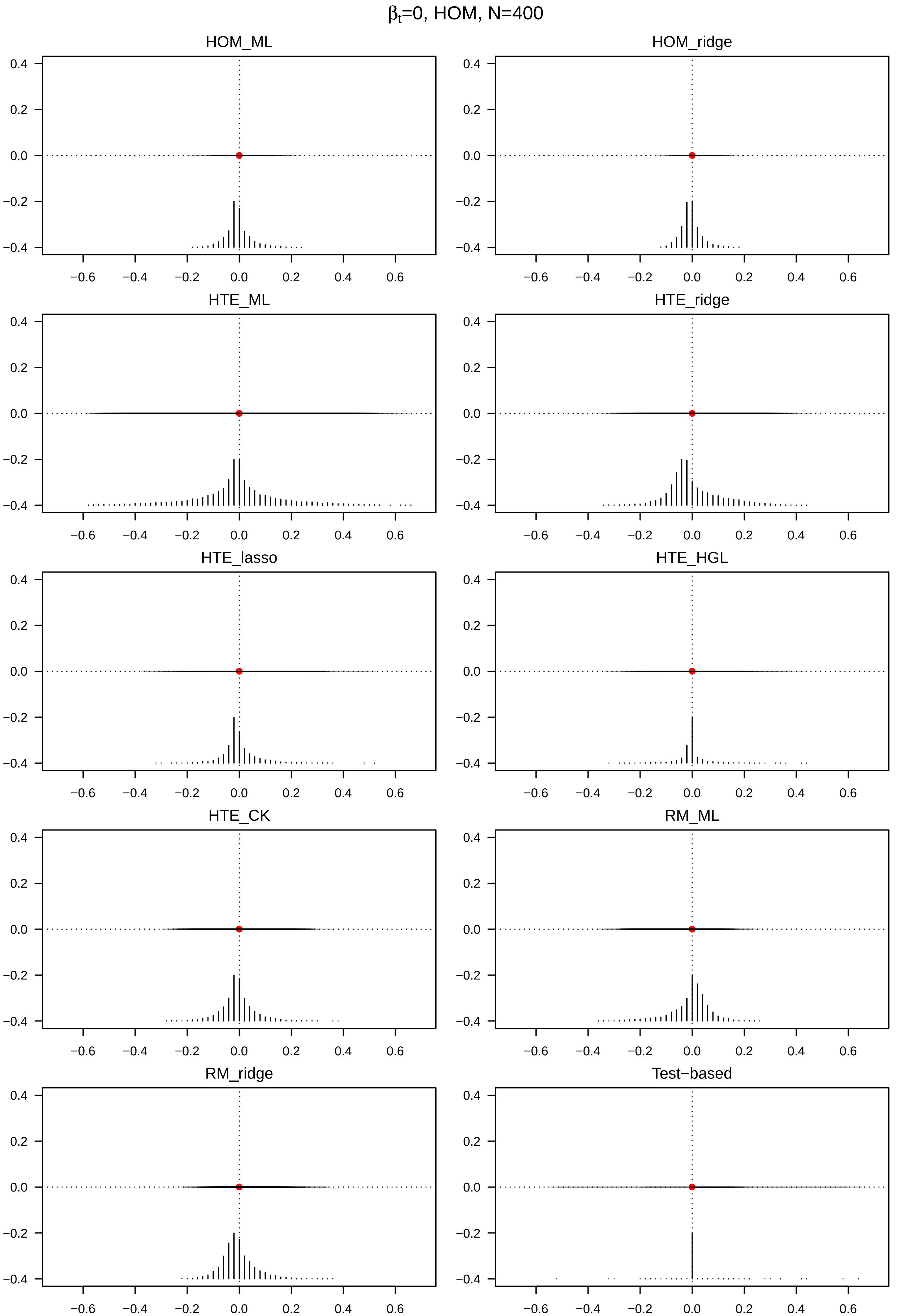
β_t=0, HOM, N=400
HOM_ML
HOM_ridge
HTE_ML
HTE_ridge
HTE_lasso
HTE_HGL
HTE_CK
RM_ML
RM_ridge
Test−based

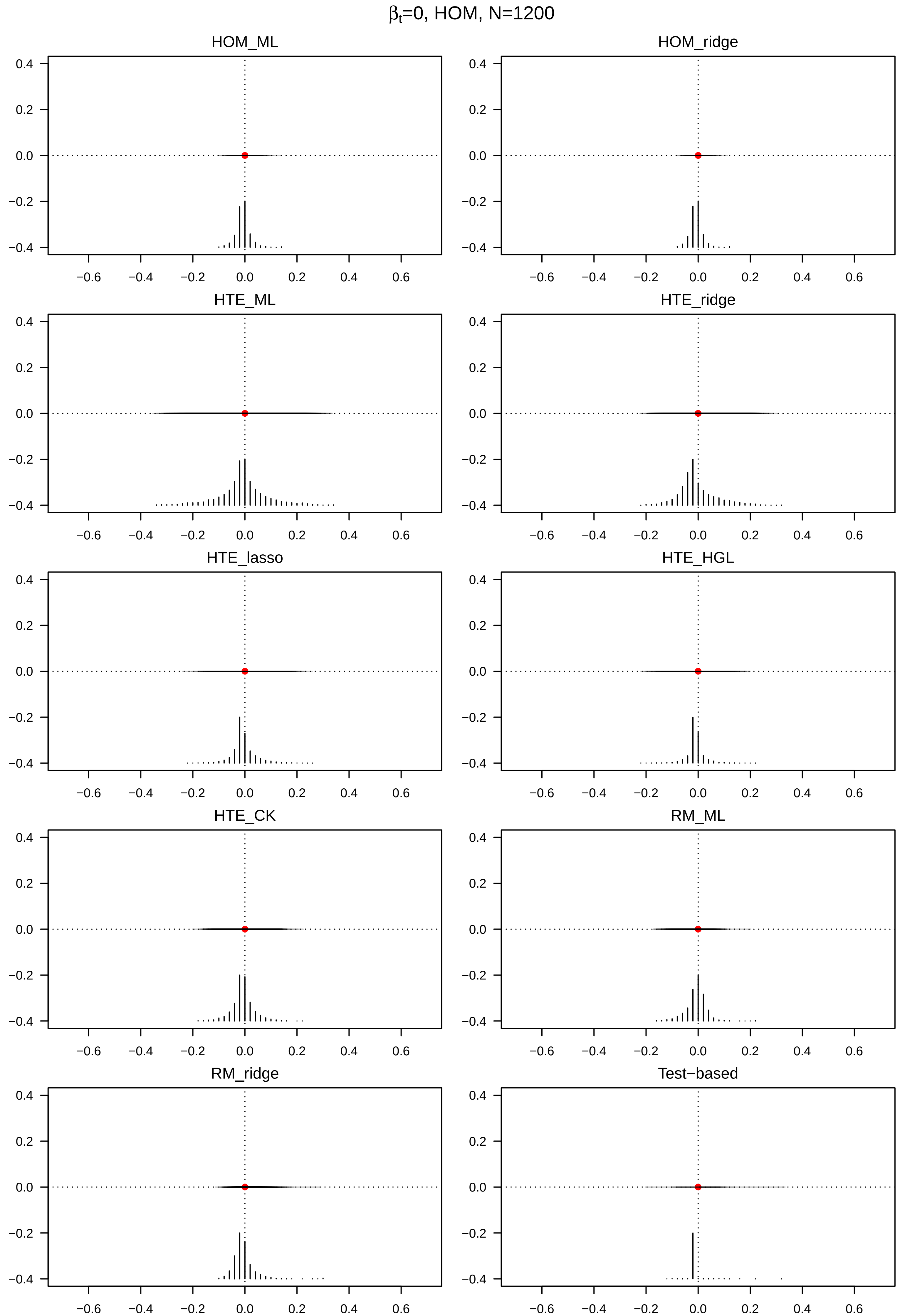

βt=0, HOM, N=1200
HOM_ML
HOM_ridge
HTE_ML
HTE_ridge
HTE_lasso
HTE_HGL
HTE_CK
RM_ML
RM_ridge
Test−based

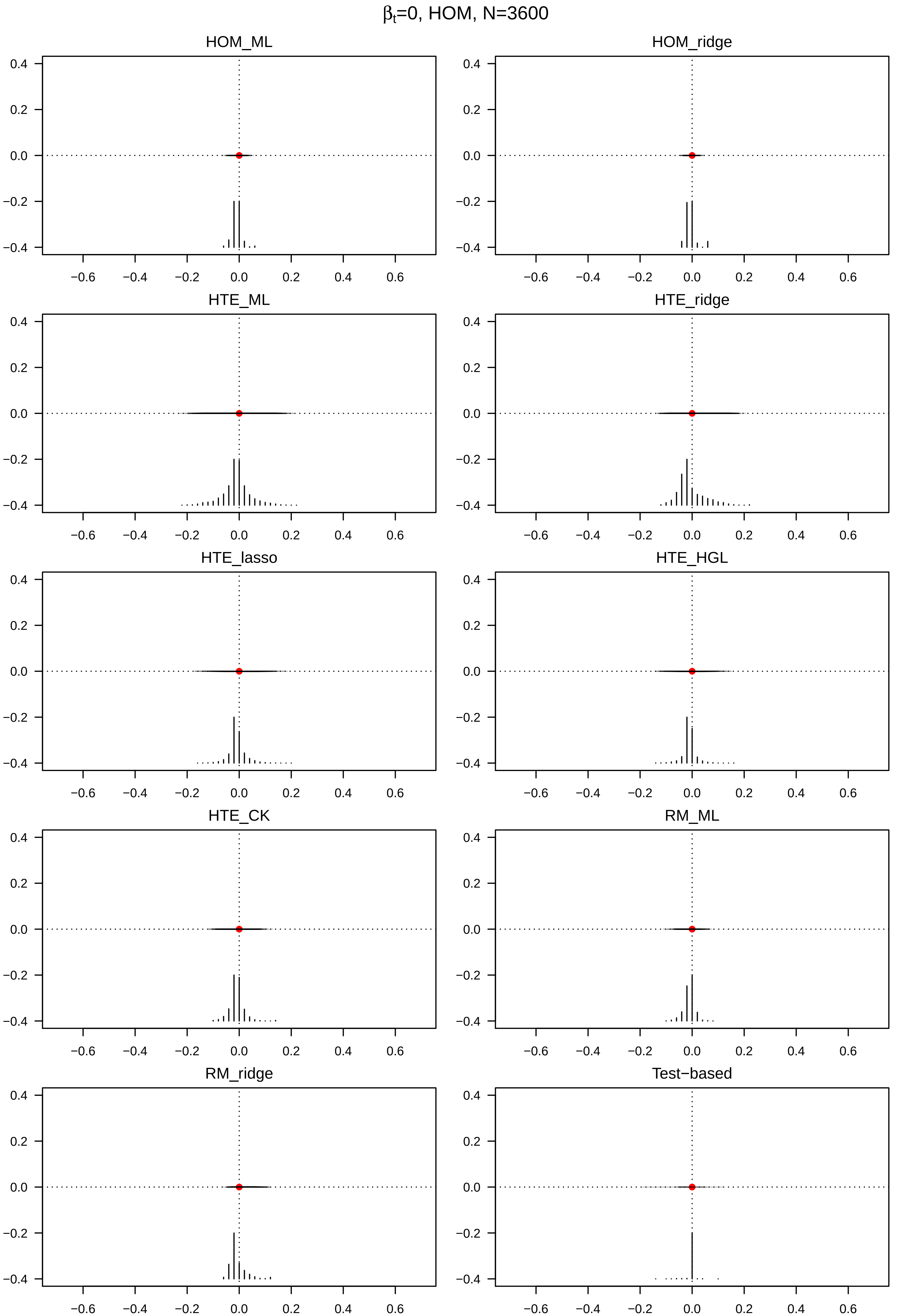

β_t=0, HOM, N=3600
HOM_ML
HOM_ridge
HTE_ML
HTE_ridge
HTE_lasso
HTE_HGL
HTE_CK
RM_ML
RM_ridge
Test−based

$\beta_t<0$, HTE, N=400

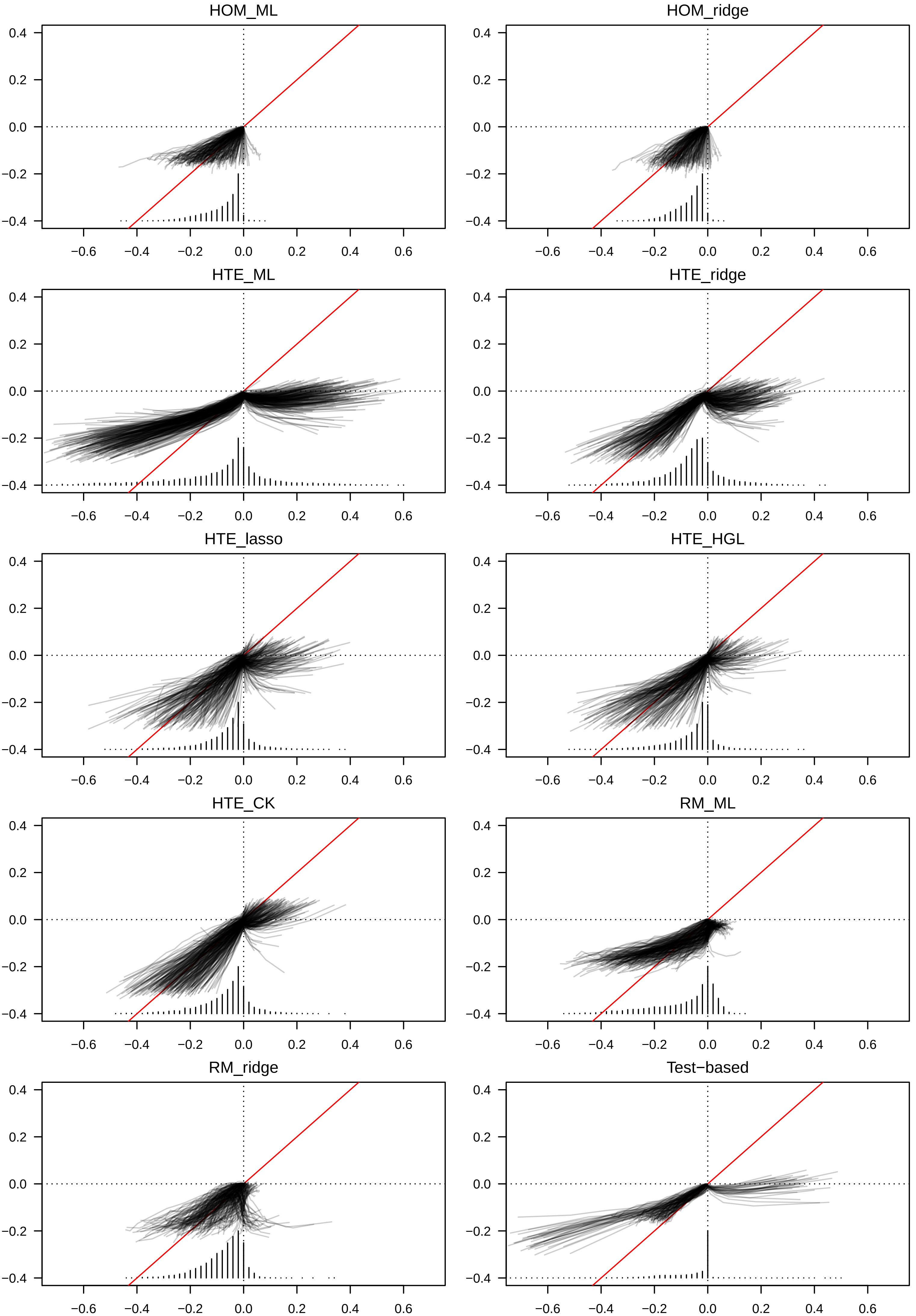

β_t<0, HTE, N=1200

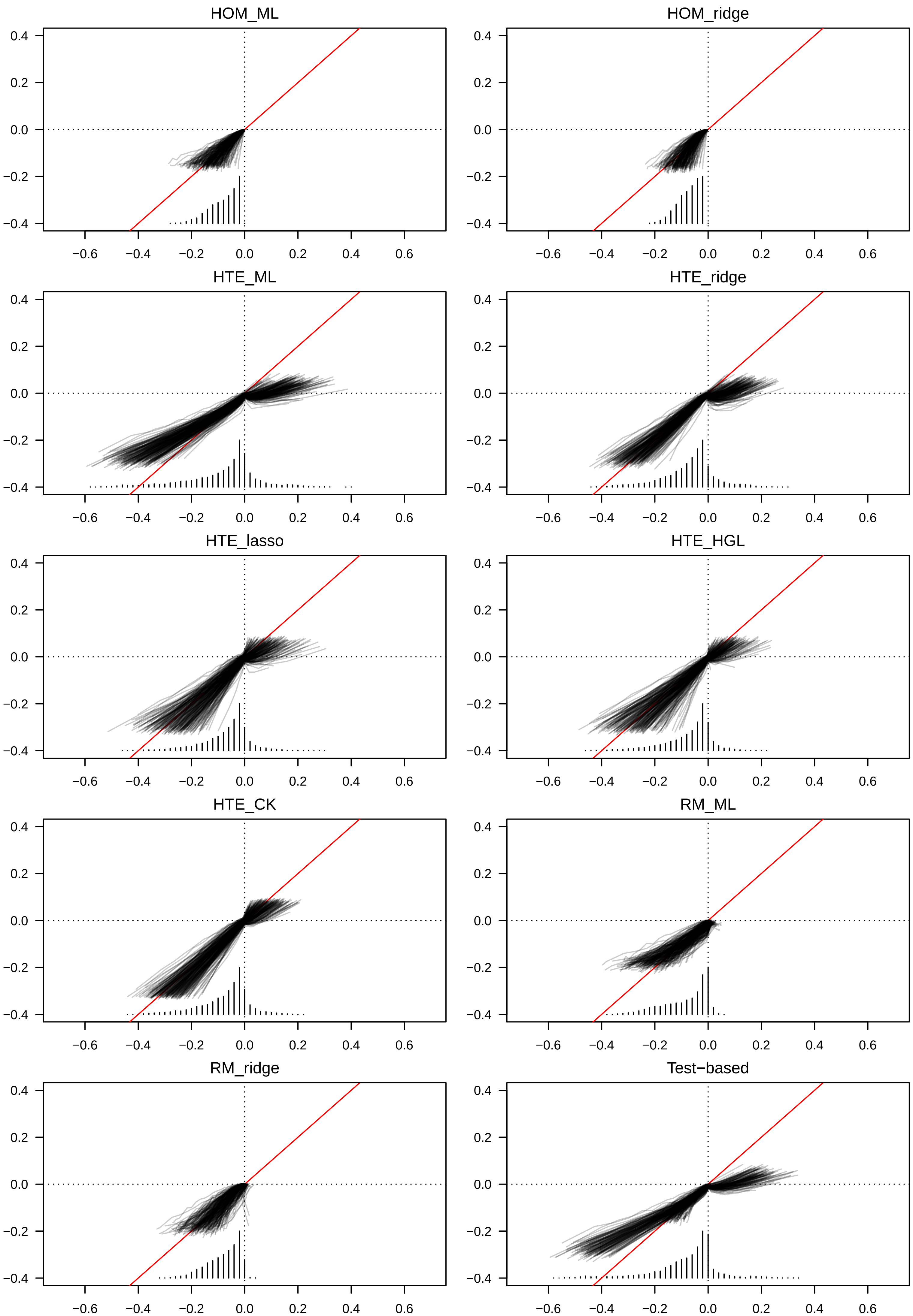

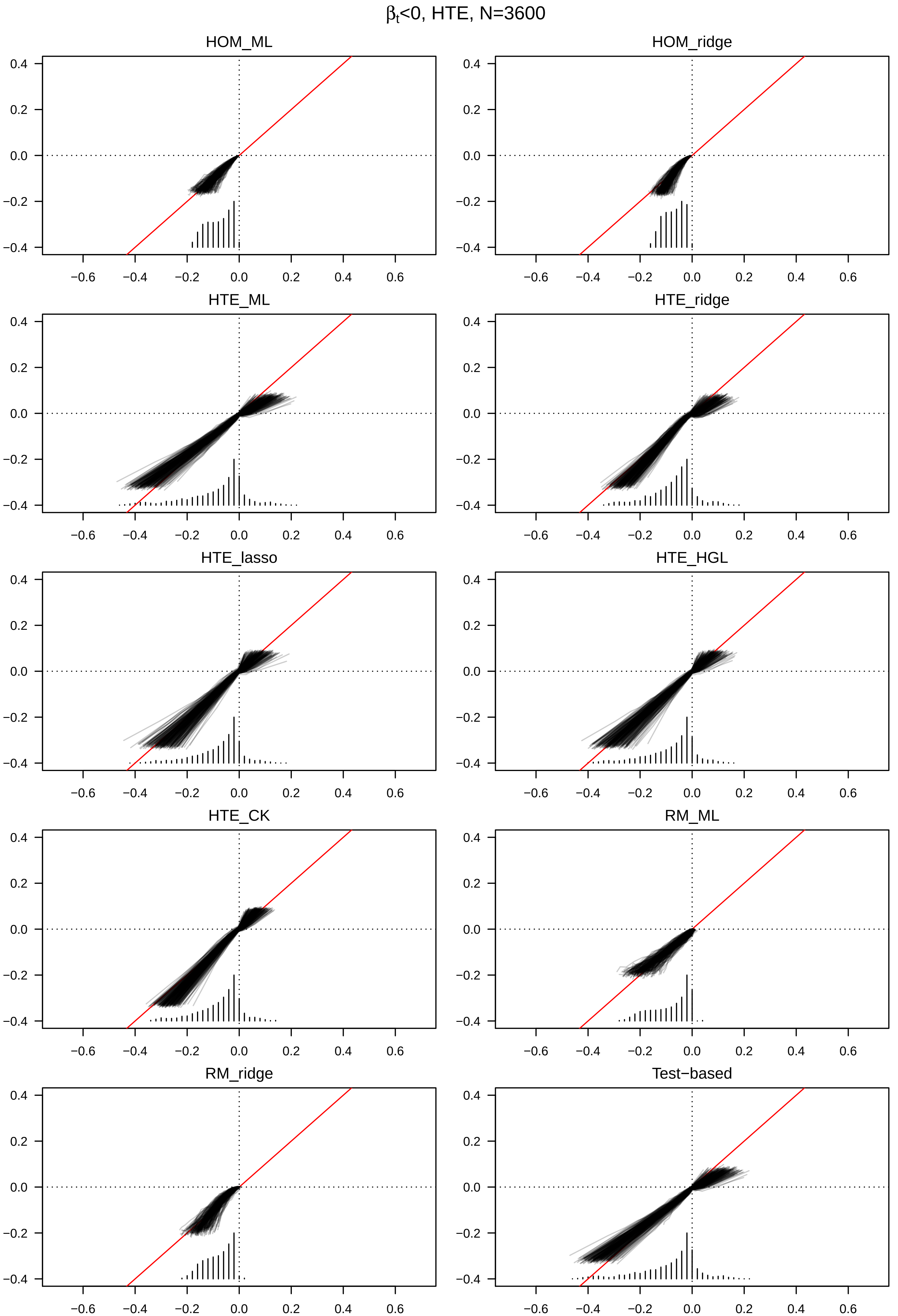
β_t<0, HTE, N=3600
HOM_ML
HOM_ridge
HTE_ML
HTE_ridge
HTE_lasso
HTE_HGL
HTE_CK
RM_ML
RM_ridge
Test−based
0.4
0.2
0.0
−0.2
−0.4
−0.6
0.6

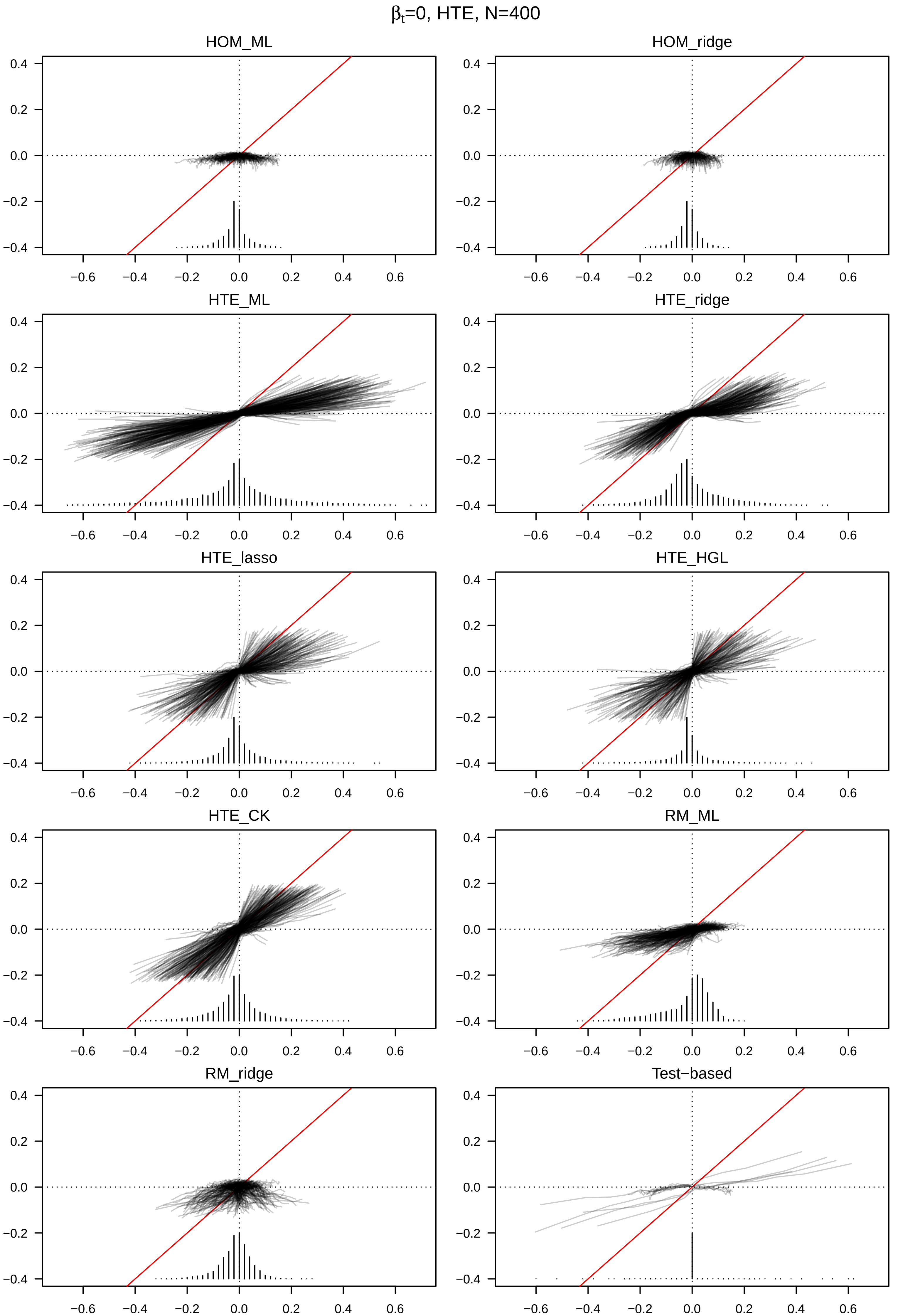

β_t=0, HTE, N=400
HOM_ML
HOM_ridge
HTE_ML
HTE_ridge
HTE_lasso
HTE_HGL
HTE_CK
RM_ML
RM_ridge
Test−based
0.4
0.2
0.0
−0.2
−0.4
−0.6
−0.4
−0.2
0.0
0.2
0.4
0.6

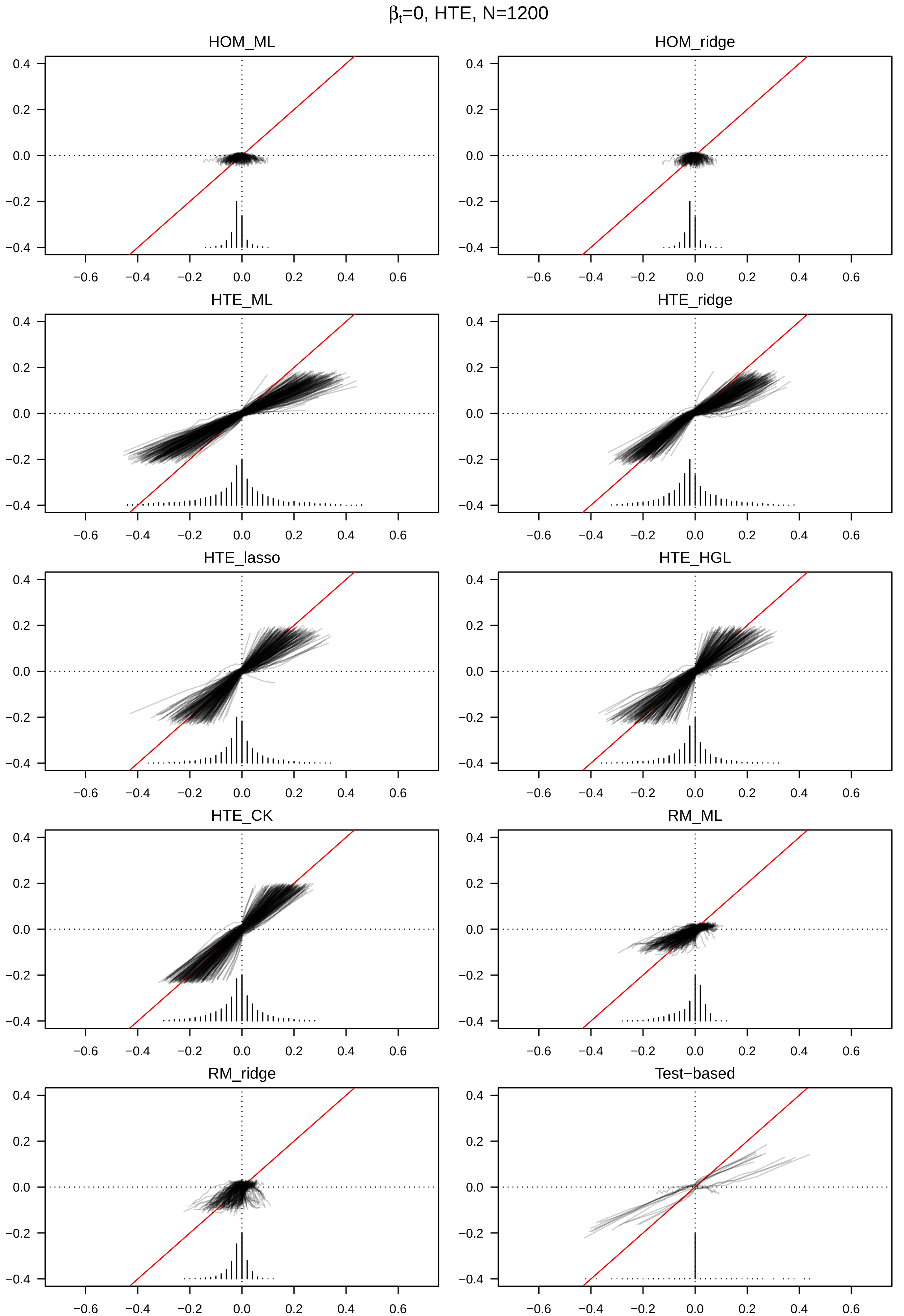

β_t=0, HTE, N=1200
HOM_ML
HOM_ridge
HTE_ML
HTE_ridge
HTE_lasso
HTE_HGL
HTE_CK
RM_ML
RM_ridge
Test−based
0.4
0.2
0.0
−0.2
−0.4
−0.6
−0.4
−0.2
0.0
0.2
0.4
0.6

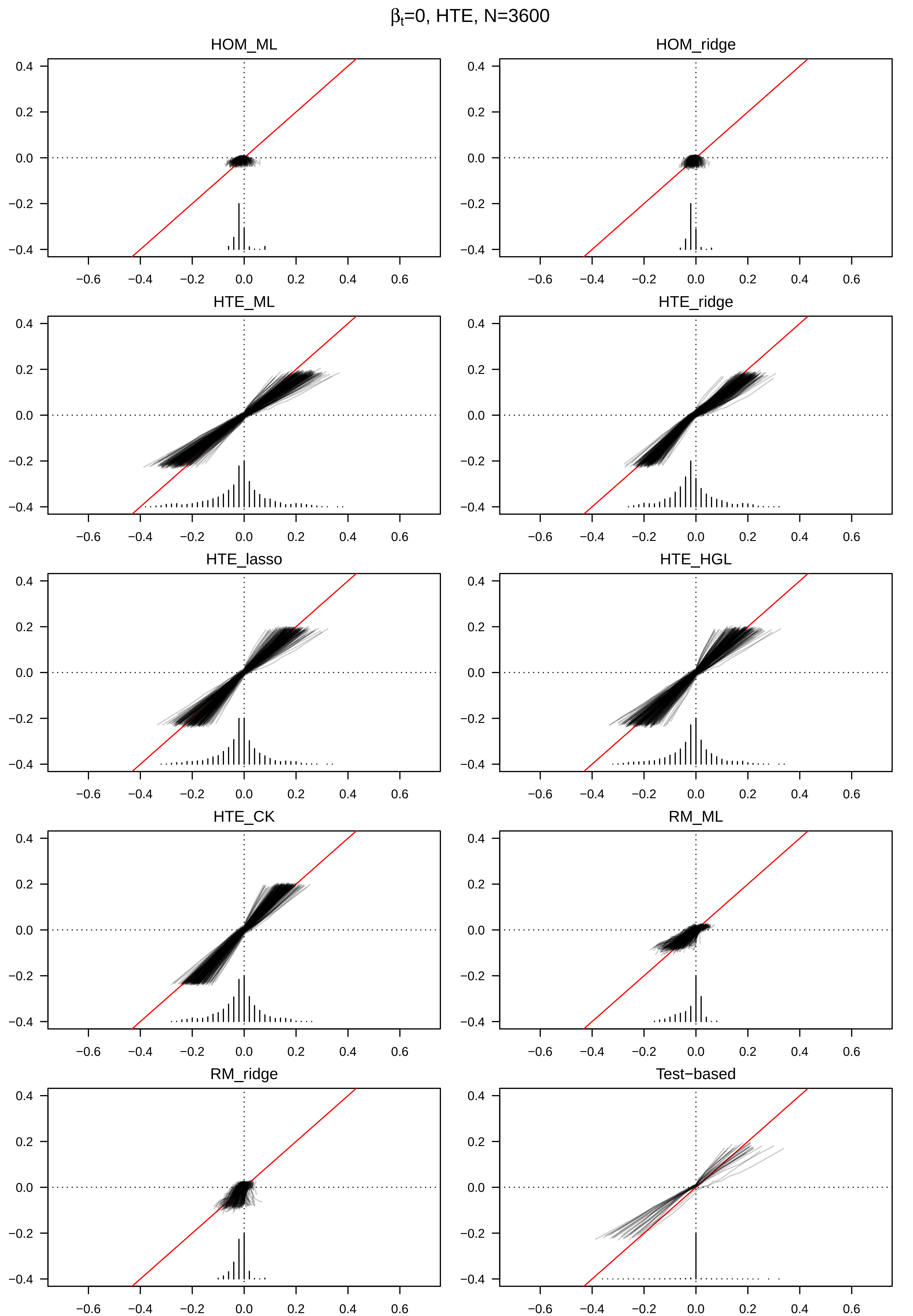

βt=0, HTE, N=3600
HOM_ML
HOM_ridge
HTE_ML
HTE_ridge
HTE_lasso
HTE_HGL
HTE_CK
RM_ML
RM_ridge
Test−based
