# Supplementary material for: A tutorial on individualized treatment effect prediction from randomized trials with a binary endpoint
Source: Stat Med. 2021 Aug 16;40(26):5961–81. doi: 10.1002/sim.9154 (PMC9291969; doi:10.1002/sim.9154)
Supplement: Supplementary file 1 — Appendix S1 Online Supporting Material [file SIM-40-5961-s001.zip › sim9154-sup-0001-supinfo.pdf]

Supporting Material

The online supporting material consists of further information on separate prediction modeling per potential outcome (Part A), the generation of data for a given outcome prevalence (Part B), and calibration results for the simulation study (Part C).

A Separate modeling of each potential outcome

This online supporting material describes the equivalence between a special case of the heterogeneous treatment effect model and models fitted separately in each arm of the trial (section A.1), the loss of this equivalence when introducing penalization (section A.2), and simulation results comparing treatment-interaction models with models fitted per treatment arm (section A.3).

A.1 Equivalent model specifications

A logistic heterogeneous treatment effect model as introduced in section 4.2 includes both main covariate effects and treatment-covariate interactions. When all covariates (or expansions thereof) in such a model interact with treatment, an exactly equivalent set of 2 models can be specified within the control group and the treated group separately. For instance, a heterogeneous treatment effect model of the form

$$\text{logit}(P(Y_i = 1|A = a_i, \boldsymbol{X} = \boldsymbol{x}_i)) = \beta_0 + \beta_t a_i + \boldsymbol{\beta}_{\boldsymbol{m}}^\top \boldsymbol{x}_i + \boldsymbol{\beta}_{\boldsymbol{z}}^\top \boldsymbol{x}_i a_i \tag{A.1}$$

has a corresponding set of within-treatment group models given by

$$\begin{aligned} \text{logit}(P(Y_i = 1|A = 0, \boldsymbol{X} = \boldsymbol{x}_i)) &= \beta_0 + \boldsymbol{\beta}_{\boldsymbol{m}}^\top \boldsymbol{x}_i \\ \text{logit}(P(Y_i = 1|A = 1, \boldsymbol{X} = \boldsymbol{x}_i)) &= (\beta_0 + \beta_t) + (\boldsymbol{\beta}_{\boldsymbol{m}} + \boldsymbol{\beta}_{\boldsymbol{z}})^\top \boldsymbol{x}_i \end{aligned} \tag{A.2}$$

Note that these two models are separate models for the potential outcomes of interest (*i.e.* $P(Y^{a=0} = 1|\boldsymbol{X} = \boldsymbol{x_i})$ and $P(Y^{a=1} = 1|\boldsymbol{X} = \boldsymbol{x_i})$ respectively). The other way around, starting from two separate models for both potential outcomes as fitted within each treatment group separately, the models

$$\begin{aligned} \text{logit}(P(Y_i = 1|A = 0, \boldsymbol{X} = \boldsymbol{x}_i)) &= \beta_{00} + \boldsymbol{\beta}_{\boldsymbol{m0}}^\top \boldsymbol{x}_i \\ \text{logit}(P(Y_i = 1|A = 1, \boldsymbol{X} = \boldsymbol{x}_i)) &= \beta_{01} + \boldsymbol{\beta}_{\boldsymbol{m1}}^\top \boldsymbol{z}_i \end{aligned} \tag{A.3}$$

are equivalent to

$$\text{logit}(P(Y_i = 1|A = a_i, \boldsymbol{X} = \boldsymbol{x}_i)) = \beta_{00} + (\beta_{01} - \beta_{00}) a_i + \boldsymbol{\beta}_{\boldsymbol{m0}}^\top \boldsymbol{x}_i + (\boldsymbol{\beta}_{\boldsymbol{m1}} - \boldsymbol{\beta}_{\boldsymbol{m0}})^\top \boldsymbol{x}_i a_i \tag{A.4}$$

The equivalence between these model specifications holds for the maximum likelihood estimates of the $\boldsymbol{\beta}$ parameter vector, but no longer holds when introducing a penalty into the estimation process.

A.2 Penalized maximum likelihood

In case of penalized maximum likelihood, estimates for the separate within treatment-group models will no longer be equivalent to those from a full sample interaction model. For instance, let us consider the case of a ridge or lasso penalty (*i.e.* $\lambda \frac{1}{2} \|\boldsymbol{\beta}\|_2^2$ or $\lambda \|\boldsymbol{\beta}\|_1$ respectively [26]). First, each of the models will have its own estimate of λ, allowing for differences between the within-treatment group models. Second, intercepts are not penalized, and in case of separate models (*e.g.* equation (A.2) and (A.3)), the main treatment effect is retrieved as the different between the two model intercepts (equation (A.4)). Hence, the main treatment effect is penalized by default in the full sample interaction model and is not penalized when using two separate models. Third, in case of ridge regression, the degree of penalization depends on the size of the model coefficients, with larger coefficients being penalized more heavily (due to the square in the penalty term). This is of importance for the treatment-covariate interaction models as specified in equation (A.1) and (A.4), since the expression of the covariate effects under treatment and control conditions is not symmetric in that case (*i.e.* with $\boldsymbol{\beta}_{\boldsymbol{m}}$ in equation (A.1) reflecting covariate effects under the control condition and $\boldsymbol{\beta}_{\boldsymbol{z}}$ reflecting changes from $\boldsymbol{\beta}_{\boldsymbol{m}}$ under the treated condition).

A.3 Simulation results

The simulation settings were exactly the same as in the main text. Full sample HTE models including all treatment-covariate interactions were compared to within-treatment group models including only main effects of the covariates. Models were estimated by means of maximum likelihood, ridge regression, and lasso regression. Figure A.1 shows the simulation study results with

respect to root mean squared prediction error (rMPSE) of the individualized treatment effects. In case of maximum likelihood estimation, the results are of course exactly the same for the different model specifications and are only shown in twofold as a reminder. Lasso treatment-covariate interaction models performed best across all settings. Also, the rMSPE of predicted individualized treatment effects based on ridge treatment-covariate interaction models was generally better than the prediction error for ridge models fitted separately per arm. One exception was ridge regression in large sample size ($N = 3600$), where the per arm models resulted in a better rMSPE. In our simulation settings, which all had variability in coefficient size in the data generating mechanism, the ridge penalty induced clear overshrinkage on large coefficients for all models. This is to be expected due to the square in the penalty and happened in both within-treatment group models and treatment-interaction models. However, in case of treatment-interaction modeling, underfitting of large main effects led to overfitting of the corresponding treatment-covariate interactions [3]. While this happened in all settings and thus across all sample sizes, we hypothesize that the negative effect of this bias on the predictions $\boldsymbol{\delta}$ was offset by more accurate estimation of λ in the full sample treatment-interaction models, except in large sample size settings. Therefore, different model specifications that affect to expected size of the estimated coefficients require careful thought in presence of a ridge penalty. These issues do no affect the lasso penalty. In case of lasso regression, the benefit of having a larger sample size to estimate the penalty parameter λ (*i.e.* as in the treatment-interaction model) led to better performance in all simulation settings.

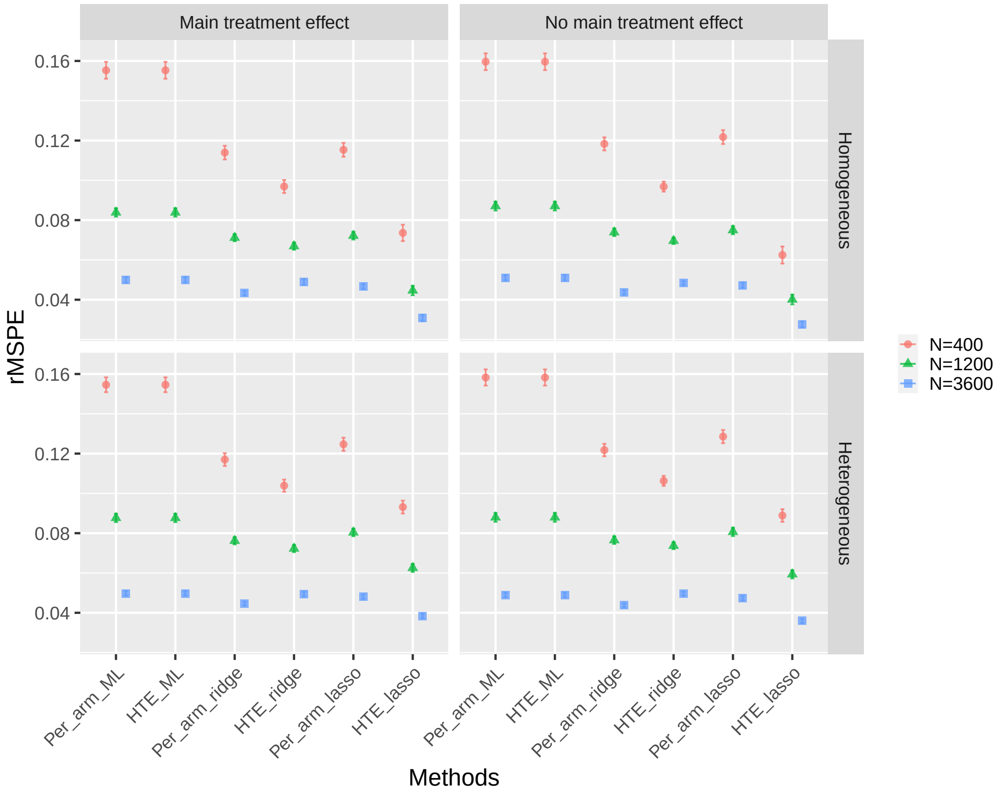

Figure A.1: Simulation study results: average root mean squared prediction error of the predicted treatment effects (over 250 simulations) with ±2 SE error bars for all simulation settings. Note that the standard errors are often so small that they are obscured by the mean estimates. Abbreviations for the methods are: HTE (heterogeneous treatment effect model), ML (maximum likelihood)

[3] Note that the square in the ridge penalty means that large estimated coefficients have a larger contribution to the penalty, and are thus more heavily penalized towards zero. An inadvertent characteristic of the treatment-interaction model in case of ridge regression is that the cost of increasing a large main effect parameter (*i.e.* in this context an increase in the effect of the covariate under the control condition), is larger than the cost of the same increase in the smaller corresponding treatment-covariate interaction (*i.e.* the same increase in the effect of the covariate but now under the treatment condition). As a numerical example, assume a main effect coefficient is actually 1 and the corresponding treatment-covariate interaction coefficient is actually 0.5. Shrinking 1 to 0.9 reduces $\|\boldsymbol{\beta}\|_2^2$ by 0.19, and overfitting 0.5 by the same amount increases $\|\boldsymbol{\beta}\|_2^2$ by only 0.11

B Simulating data for a given outcome prevalence

The goal was to simulate data with a prespecified outcome prevalence for the control group. The model underlying the simulations was given in equation (7) and is restated here for ease of reference:

$$\text{logit}(P(Y_i = 1|A = a_i, \boldsymbol{X} = \boldsymbol{x}_i)) = \beta_0 + \beta_t a_i + \boldsymbol{\beta}_{\boldsymbol{m}}^\top \boldsymbol{x}_i + \boldsymbol{\beta}_{\boldsymbol{z}}^\top \boldsymbol{z}_i a_i \tag{B.1}$$

For any given treatment condition, this reduces to

$$\text{logit}(P(Y_i = 1|A = a_i, \boldsymbol{X} = \boldsymbol{x}_i)) = \beta_{0*} + \boldsymbol{\beta}_{\boldsymbol{*}}^\top \boldsymbol{x}_i \tag{B.2}$$

where β_{0*} combines β_0 and β_t and $\boldsymbol{\beta}_{\boldsymbol{*}}$ combines $\boldsymbol{\beta}_{\boldsymbol{m}}$ and $\boldsymbol{\beta}_{\boldsymbol{z}}$. Therefore, conditional on treatment condition, the log odds of an event is a linear combination of just the p covariates. Since these had a standard normal distribution by design, their linear combination is also normal with mean equal to β_{0*} and variance equal to

$$\text{Var}(\beta_{0*} + \boldsymbol{\beta}_{\boldsymbol{*}}^\top \boldsymbol{x}_i) = \boldsymbol{\beta}\boldsymbol{\Sigma}\boldsymbol{\beta}^\top = \sigma^2 \tag{B.3}$$

where $\beta = \{\beta_{0*}, \boldsymbol{\beta}_{\boldsymbol{*}}\}$ and $\boldsymbol{\Sigma}$ is the covariance matrix of the covariates.

Then using

$$\Pr(Y = 1) = \frac{1}{1 + e^{-\beta_{0*} - \sigma Z}} \tag{B.4}$$

where Z is a standard normally distribute random variable, the outcome prevalence or expected probability of $\Pr(Y = 1)$ equals

$$\begin{aligned} \mathbb{E}(\Pr(Y = 1|\boldsymbol{X})) &= \int_{-\infty}^{+\infty} \left(\frac{1}{\sqrt{2\pi}} e^{-z^2/2} \frac{1}{1 + e^{-\beta_{0*} - \sigma Z}} \right) dz \\ &= \frac{1}{\sqrt{2\pi}} \int_{-\infty}^{+\infty} \left(\frac{e^{-z^2/2}}{1 + e^{-\beta_{0*} - \sigma z}} \right) dz \end{aligned} \tag{B.5}$$

Since $\sigma = \sqrt{\boldsymbol{\beta}\boldsymbol{\Sigma}\boldsymbol{\beta}^\top}$ only depends on known simulation parameters, the equation can be solved numerically for β_{0*} to get the desired outcome prevalence in a given treatment group.

C Simulation study calibration results

The `CalibrationFigures.pdf` file contains calibration plots for $\hat{\delta}(\boldsymbol{x}_i)$, as predicted by each method, versus the true $\delta(\boldsymbol{x}_i)$. Simulation settings with a main treatment effect are denoted as $\beta_t < 0$, settings with a homogeneous treatment effect are denoted as HOM, and settings with a heterogeneous treatment effect as HTE. Each individual plot shows the ideal diagonal in red (with an exception of the absolute null settings where the ideal is $\hat{\delta}(\boldsymbol{x}_i) \equiv 0$). Each black calibration line is the result of a single simulation run and connects the mean predicted $\hat{\delta}(\boldsymbol{x}_i)$ and mean $\delta(\boldsymbol{x}_i)$ in 20 equal-size quantile groups of $\hat{\delta}(\boldsymbol{x}_i)$. The histograms on the x-axis gives an indication of the density of quantile groups over all simulation (the groups vary due to sampling variability).
